# Randomized, placebo controlled, double blinded pilot superiority phase 2 trial to evaluate the effect of curcumin in moderate to severe asthmatics

**DOI:** 10.1186/s12890-021-01619-y

**Published:** 2021-08-17

**Authors:** Michele Quan, Abdullah Alismail, Noha Daher, Derrick Cleland, Sonia Chavan, Laren D. Tan

**Affiliations:** 1 Department of Medicine, School of Medicine, Loma Linda University Health, Loma Linda, CA USA; 2 Department of Cardiopulmonary Sciences, School of Allied Health Professions, Loma Linda University Health, Loma Linda, CA USA; 3 Allied Health Studies, School of Allied Health Professions, Loma Linda University Health, Loma Linda, CA USA; 4 La Sierra University, Riverside, CA USA; 5 Division of Pulmonary, Critical Care, Hyperbaric, Allergy and Sleep Medicine, Loma Linda University Health, 11234 Anderson Street, Suite 6439, Loma Linda, CA 92354 USA

**Keywords:** Asthma, Curcumin, Anti-inflammatory, Anti-oxidant, Severe asthma, Moderate asthma

## Abstract

**Background:**

Curcumin, a derivative of the spice turmeric, has been adopted by Eastern medicine for centuries as an adjunct to treat several medical conditions (e.g., anorexia and arthritis) because of its well-established anti-inflammatory properties. Studies have shown that the use of curcumin in mice models has led to reduction in several inflammatory markers as well as key inflammatory pathway enzymes. As a result, studies in Western medicine have developed to determine if this recognized benefit can be utilized for patients with inflammatory lung diseases, such as asthma. This study will seek to better understand if curcumin can be used as an adjunctive therapy for improving asthma control of patients with moderate to severe asthma; a finding we hope will allow for a more affordable treatment.

**Methods:**

This study will utilize a randomized, placebo controlled, double blinded pilot superiority phase 2 trial at an outpatient pulmonary clinic in Southern California, USA. Subjects will be receiving Curcumin 1500 mg or matching placebo by mouth twice daily for the study period of 12 weeks. Subjects will be randomized to either a placebo or intervention Curcumin. Subjects will have 6 clinic visits: screening visit, a baseline visit, monthly clinic visits (weeks 4, 8, and 12), at weeks 4, 8, and a follow-up clinic visit or phone-call (week 16). Changes in asthma control test scores, number of days missed from school/work, FEV1 (% predicted), FEV1/FVC ratio, FVC (% predicted), blood eosinophil count, blood total IgE, and FeNO levels will be compared by group over time.

**Discussion:**

The therapeutic effects of curcumin have been studied on a limited basis in asthmatics and has shown mixed results thus far. Our study hopes to further establish the benefits of curcumin, however, there are potential issues that may arise from our study design that we will address within this paper. Moreover, the onset of the COVID-19 pandemic has resulted in safety concerns that have delayed initiation of our study. This study will contribute to existing literature on curcumin’s role in reducing lung inflammation as it presents in asthmatics as well as patients suffering from COVID-19.

***Trial registration*:**

This study protocol has been approved by the Institutional Review Board at Loma Linda University Health, (NCT04353310). IND# 145101 Registered April 20th, 2020. https://clinicaltrials.gov/ct2/show/NCT04353310.

## Background

Curcumin, a derivative of turmeric (*Curcuma longa*), has been commonly used as a dietary spice and coloring agent. Furthermore, it has been used for centuries in Eastern medicine has been used for centuries in Ayurveda and traditional Chinese medicine to treat anorexia, hepatic disorders, and arthritis [1]. The biologic effects of curcumin include anti-inflammatory, anti-oxidant and anti-neoplastic, mediated by molecular targets including transcription factors, inflammatory cytokines, and proteins involved in cell replication and survival [2]. In vitro and in vivo models have shown that curcumin’s anti-inflammatory effect is mediated by nuclear factor kappa B (NF-κB), a transcription factor that regulates the expression of several genes that are involved in both innate and adaptive immunity and inflammation [3]. Additionally, more recent studies in vitro and in vivo mice models have also discovered that curcumin inhibits cyclooxygenase-2 (COX-2), a key enzyme involved in the development of prostaglandins [4]. Furthermore, it has been shown to inhibit the secretion of proinflammatory Tumor Necrosing Factor-alpha (TNF-α) and Interleukin-6 (IL-6) and anti-inflammatory (IL-10) cytokines [5]. In addition, curcumin has been found to decrease the release of eotaxin, monocyte chemotactic protein 1, and monocyte chemotactic protein 3 from IL-1β stimulated human airway smooth muscle cells [6].

Moreover, when curcumin is added to Dermatophagoides farinae (Der f)–stimulated lymphocyte cell cultures from allergic asthmatic patients, curcumin inhibits Der f– induced lymphocyte proliferation and production of interleukins (IL-2, IL-4, & IL-5), and granulocyte macrophage colony- stimulating factor [7]. It has also be reported that curcumin has an effect on the airways by decreasing the constriction and hyperreactivity when it is administered with ovalbumin [8, 9].

**Fig. 1:**
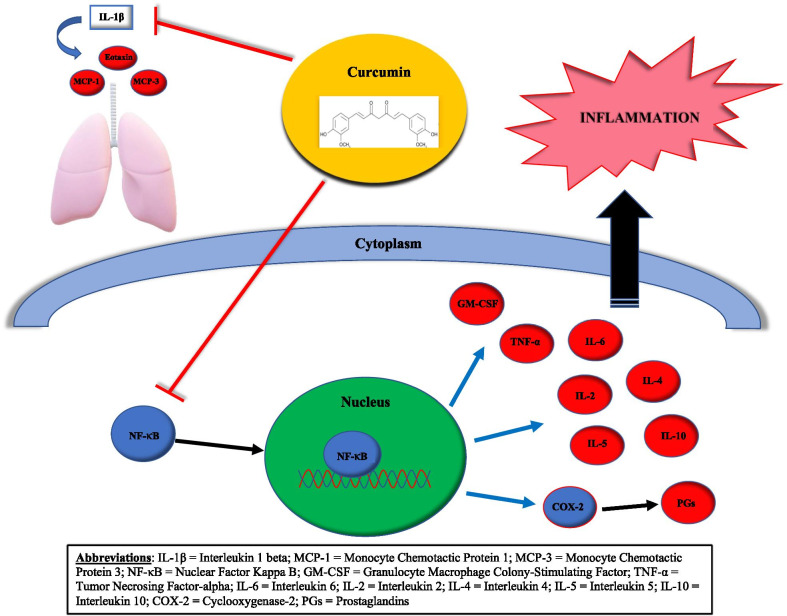
Diagram that illustrates the mechanism of action of curcumin and the biologic effect

A review of data regarding the biological effects of curcumin in asthma found that most data has been acquired in vitro or in vivo animal models, with only a few observational studies available in humans with discordant results [10]. In one study with mice, inhibition of NF-κB activity reduced airway hyper-responsiveness and inflammatory cell airway infiltration. This was most effective via intraperitoneal administration, and oral administration of curcumin only produced minimal improvement in comparison to controls [11]. In another study with mice, it was shown that curcumin activated the nuclear factor erythroid 2-related factor 2/heme oxygenase 1 (Nrf2/HO-1) signaling pathway in a dose and time-dependent manner, with resulting decline in TNF-α, IL-1β, and IL-6 levels in vitro. Additionally, it was found to decrease eosinophil and white blood count (WBC) in bronchoalveolar lavage, and airway hyper-responsiveness [12], Fig. 1.

Kim et al. studied a daily dose of 2000 mg based on a previous study by Baum et al. (2008) in patients with Alzheimer [9, 13]. Kim et al. (2011) found that curcumin did not significantly affect in various outcomes such as postbronchodilator Forced Exhaled Volume in 1 s (FEV1), Asthma Control Test scoring, use of rescue bronchodilator, dose of inhaled corticosteroid, exhaled nitric oxide, serum total IgE, or blood eosinophils [9].

Abidi et al. conducted a randomized control trial on 77 patients with mild to moderate bronchial asthma with documented positive bronchodilator reversibility [14]. Curcumin capsules improved airway obstruction evidenced by significant improvement in mean FEV1 values and hematological parameters [14].

The *purpose* of this study will be to identify an adjunctive therapy that is both cost-effective and safe for patients suffering from moderate to severe asthma so as to limit the need for adding more costly medicinal therapies, such as biologics. The *objective* will be to evaluate the effects of oral curcumin supplementation in adult patients with moderate to severe asthma. We *hypothesize* that high-dose supplementation with curcumin will lead to fewer exacerbations and improved control among asthmatics.

## Methods and design

This study will be a randomized, placebo controlled, double blinded pilot superiority phase 2 trial. The rationale for choosing a randomized, placebo control trial is to avoid bias in patient allocation-to-treatment and increase the probability that any differences between groups can be attributed only to the treatment. This study is a pilot study given that there is limited to no data that currently demonstrates curcumin as an effective treatment for asthma.

Recruited subjects will undergo a screening process to ensure they meet the following study criteria listed in Table 1.

**Table 1:** Inclusion and exclusion criteria for the study

Inclusion criteria	Exclusion criteria
Male or female, aged 18 and older Physician diagnosed moderate to severe asthma: (GINA 2018) Stable asthma that requires ICS/LABA and/or an additional controller agent (i.e. LTRA, LAMA) Ability to take oral medication and be willing to adhere to the regimen Ability to speak and read English If female and sexually active, should use effective forms of birth control	Current use of turmeric (curcumin) or use within the last 7 days Current use of biologic therapy/immunotherapy/ or bronchothermoplasty Pregnancy or lactation Known allergic reactions to components of turmeric (curcumin) Current use of anticoagulants, and history of coagulopathy or liver disease INR greater than 2.0, PTT greater than 45.0 s

*GINA* Global initiative for asthma, *ICS* inhaled corticosteroids, *LABA* long-acting beta agonist, *LTRA* leukotriene receptor antagonist, *LAMA* long acting muscarinic, *INR* international normalized ratio, *PTT* partial thromboplastin time

All subjects will be screened to ensure meeting inclusion criteria. Screening failure is attained when a participant consents to participate but is not subsequently randomly assigned to either the study intervention or control. In addition, any individual who does not meet the criteria for participation due to meeting exclusion criteria (screening failure) maybe rescreened. Rescreened participants will be assigned the same participant number as for the initial screening.

### Study location

One site, outpatient pulmonary academic center in Southern California, United States of America.

### Recruitment strategy

Multiple methods will used to recruit participants in the study. First, flyers will be posted in the medical offices for patients to see. Clinic staff, nursing, and physicians will recruit and identify potential participants. In addition, to ensure meeting NIH policy on inclusion of women and minorities, we anticipate recruiting men, women, and minority groups in equal fashion as indicated in the inclusion criteria (all adults with moderate to severe asthma).

### Design

After screening and meeting inclusion and exclusion criteria, subjects will be randomized 1:1 to one of two arms: (1) placebo (n_1_ = 15) or (2) intervention (n_2_ = 15), curcumin. Once assigned into their group, subjects will receive double-blinded study medication to take daily for 12 weeks. In total, subjects will have up to 6 clinic visits: screening visit (D-7 to -1), a baseline visit (V1), three monthly clinic visits (V2-4) and a follow-up clinic visit or phone-call (week 16).

### Randomization and blinding

Group assignment will be conducted by an independent person (unblinded IDS pharmacist and technician). The unblinded IDS pharmacist will assign the next sequential randomization number assignment from a computer-generated list and dispense assigned study drug in a blinded fashion. This randomization list will be maintained by IDS and not be shared with anyone on the blinded investigator team or blinded subjects. In case of emergency, the study principal investigator, based on clinical judgment, may request the IDS pharmacist to unblind an individual subject’s treatment assignment. Plans to unblind the investigators will occur at the end of the study when data analysis is completed.

### Dosing and administration

Subjects will be receiving curcumin 1500 mg or matching placebo by mouth twice daily for the study period, 12 weeks. Any missed or vomited doses will be skipped and resumed at the next scheduled time. Subjects will use a dosing calendar to record administration or missed doses. The reason for selecting this dose is that curcumin (at 20 mg kg^−1^ bodyweight) was reported to attenuate allergen-induced airway hyper-responsiveness in sensitized guinea pigs [8]. It has been reported that humans can tolerate a dose of curcumin as high as 12 g/day, without any toxic side effects [15]. Therefore, we hypothesize that a dose of 3000 mg curcumin daily may significantly improve asthma control and affect other biomarkers of inflammation.

Sabinsa corporation will be providing the study drugs, both curcumin and matching placebo. Curcumin formulation will include 95% curcuminoids with black pepper extract plus inert excipients. The placebo formulation will contain the same inactive excipients found in curcumin. Both formulations will be identical in appearance. All study drugs will be clearly labeled for use for only this protocol and will not be shared or used for any patients not enrolled in this protocol. All study drug labels will include, *“Caution: New Drug Limited by Federal Law to Investigational Use."* Upon dispensing study drug will be labeled for subject specific use to be administered at home as prescribed by the investigator. All outpatient labels will follow California state Pharmacy laws for prescription drugs and be identified in a blinded fashion.

Study drugs will be stored at room temperature in their original containers for the length of time as instructed by the manufacturer Sabinsa as per their stability testing. Study drugs will be stored per Federal regulations, securely in limited-access pharmacy area and separately from commercial products. Storage temperature will be monitored daily by IDS to ensure proper storage is maintained.

### Concomitant therapy

Current background standard of care treatment for asthma will continue during the study period. Medications to be reported in the Case Report Form are concomitant prescriptions medications, over-the-counter medications, and supplements. Any newly prescribed medications, over the counter or supplements are allowed except for curcumin. Concomitant therapy may affect the outcome as it can alter lab values and perceived side effects.

### Discontinuation of study intervention

If the subject discontinued using curcumin, this will translate to discontinuation from the study, and remaining study procedures will follow as indicated by the protocol. If a clinically significant finding is identified by the study investigators (including, but not limited to changes from baseline) after enrollment, the investigator or qualified designee will determine if any change in participant management is needed. Any new clinically relevant finding will be reported as an adverse event (AE).

The data to be collected at the time of study intervention discontinuation will include the following: (1) reason for discontinuation of medication, (2) any side effects or symptoms experienced, and (3) cumulative dosage of curcumin ingested at that time.

In addition, participants are free to withdraw from the study at any time upon request, the study investigator may also discontinue or withdraw the participant from the study if any of the following occurred: (1) significant study intervention non-compliance, (2) if any clinical adverse event (AE), laboratory abnormality, or other medical condition or situation occurs such that continued participation in the study would not be in the best interest of the participant, (3) disease progression which requires discontinuation of the study intervention, (4) if the participant meets an exclusion criterion (either newly developed or not previously recognized) that precludes further study participation, and (5) participant is unable to receive curcumin tablets for 12 weeks. Study investigators will document these reasons as Case Report Form.

### Replacements

Subjects will be replaced with other subjects under the following conditions: (1) if they signed an informed consent form, randomized but did not receive study intervention; (2) if they signed the informed consent from, randomized, received the study intervention, and subsequently withdrew or withdrawn/discontinued from completing the study.

### Lost to follow up

A subject is lost to follow up if they fail to return for scheduled visits and/or unable to be contacted and reached by the study site staff. We anticipate taking several additional and action measures when lost to follow up occurs such as counseling the subjects on the importance of the scheduled visit, willingness to continue and be part of the study, multiple telephone calls by the investigators, and mailing a letter to the last known address on file.

## Detailed Study procedure

### Baseline

After the subject(s) sign the informed consent, they will undergo history, physical exam including height and weight specifically cardiopulmonary examination, vital signs (blood pressure, heart rate, respiratory rate, and oxygen saturation), and an initial questionnaire that will include current asthma control test and demographics [16, 17]. Subjects will undergo randomization and will then be provided the curcumin or placebo. One member of the study staff will be responsible for randomization but will not share information regarding randomization with the subject nor study staff. Subject will undergo spirometry testing in the clinic site conducted by an experienced respiratory therapist or physician. Effort acceptance criteria is appropriate (American Thoracic Society Grade A—prefer 3 separate efforts with two largest FVC (Forced Vital Capacity) measurements with agreement within 150 mL, and FEV1 (Forced Expiratory Volume in the first second) agreement within 150 mL, B is acceptable if that is the best result after 8 efforts). Spirometer is handheld, and a calibration check will be performed daily with a 3L syringe prior to testing. In addition, Fraction of Exhaled Nitric Oxide (FeNO) will be collected and measured using NIOX VERO® Sensor machines.

Laboratory work will be obtained at the initial visit for plasma eosinophil count and serum total IgE, as well as baseline INR and PTT. Plasma eosinophil count will be drawn in approximately 1.5 mL of blood collected in a lavender top tube. IgE level is minimum 500 µL of blood, although 2 mL is preferred, collected in red tubes (serum) or light green tubes (lithium heparin). Plasma INR/PT and PTT will be collected in 3 mL blue-top tubes. Laboratory specimens will be analyzed via the Loma Linda University Medical Center Clinical Laboratory in compliance with the Clinical Laboratory Improvement Amendments (CLIA) of 1988. Urine pregnancy tests will be conducted for all applicable participants (i.e. females in reproductive age who are not currently on birth control) at baseline, and again at 4, 8 and 12 weeks.

### Visits 2 and 3

During subsequent visits (Weeks 4, 8), subjects will fill out a questionnaire that includes the Asthma Control Test which evaluates the number of severe asthma exacerbations patient has had including: worsening of symptoms for more than or equal to 48 h of consecutive bronchodilator use, any hospitalizations or emergency room visits [16, 17]. The questionnaire will also evaluate the number of missed days from school or work patient has had from asthma. Subjects will undergo spirometry testing in clinic which will be conducted by an experienced respiratory therapist or physician. Exhaled nitric oxide will be measured at that time as well.

### Visit 4

Subjects will fill out a questionnaire that includes the Asthma Control Test, evaluates the number of severe asthma exacerbations patient has had including worsening of symptoms for more than or equal to 48 h of consecutive bronchodilator use, any hospitalizations or emergency room visits. The questionnaire will also evaluate the number of missed days from school or work patient has had from asthma. Participant will undergo spirometry in clinic which will be conducted by an experienced respiratory therapist or physician. Exhaled nitric oxide will be measured at that time. Laboratory work will be obtained at this initial visit to measure plasma eosinophil count and serum total IgE.

### Follow up

At week 16, the subject will be called or will visit the clinic to evaluate for any adverse effects of the drug. For subjects that may discontinue or withdraw early, we will record the rationale on final visit or via telephone conversation and cumulative curcumin dosage at that time point.

Table 2 shows the schedule of activities subjects will undergo in this study from beginning to end. Once the subject complete all phases of the study, indicated in Table 2, they are considered as completed the study.

**Table 2:** Schedule of Activities that study subjects will go through from beginning to end

Procedures	Screening Day-7 to -1	Enrollment/baseline Visit 1, Day 1	Study visit 2 Week 4	Study visit 3 Week 8	Study visit 4 Week 12	Follow up Week 16
Informed consent	X					
Demographics	X					
Medical history	X					
Randomization		X				
Administer study intervention		X				
Concomitant medication review	X	X	X	X	X	
Physical exam (including height and weight)	X	X			X	
Vital signs	X	X			X	
ACT Scoring/ Questionnaire		X	X	X	X	
Spirometry (FVC, FEV1)		X	X	X	X	
Blood studies (eosinophils, IgE)		X			X	
Exhaled nitric oxide		X	X	X	X	
INR and PTT		X				
Urine pregnancy test (*if applicable)*		X	X	X	X	
Adverse event review and evaluation		X	X	X	X	X
Complete Forms	X	X	X	X	X	X

*ACT* Asthma control test, *FVC* forced vital capacity, *FEV1* forced expiratory volume in 1 s, *IgE* immunoglobulin E, *INR* international normalized ratio, *PTT* partial thromboplastin time

### Data analysis

Assuming a 30% attrition rate, a sample size of 30 participants is estimated using a moderate effect size for the group x time interaction (f = 0.25 or partial η^2^ = 0.06), level of significance (α = 0.05), and power of 0.80. Effect size was calculated using GPower software (version 3.1.2, University of Dusseldorf, Dusseldorf, Germany).

Data will be summarized using mean and standard deviation for quantitative variables and counts (%) for qualitative variables. The normality of continuous variables will be examined using Shapiro Wilk’s test. The distribution of the participants’ characteristics by study group will be evaluated using chi-square for qualitative variables and independent t- test for quantitative variables. A 2-groupX 4-time points (baseline, week 4, week 8, and week 12) mixed factorial analysis of variance (ANOVA) will be used to examine changes in asthma control test scores, number of days missed from school/work, FEV1 (% predicted), FEV1/FVC ratio, FVC (% predicted), blood eosinophil count, blood total IgE, and FeNO levels by group over time.

There will two main analyses that will be conducted in this study. First: a comparison between study groups will be conducted by looking at group x time interaction effect. The interaction will be evaluated and if there is a statistical significance, an independent t-test will be used by comparing change from baseline between groups at each follow up. A post-hoc Bonferroni test will be conducted for the combined groups if the main effect of time in the mixed factorial found to be significant. Second: A one-way repeated measures, ANOVA, will be used for within-groups to test changes from baseline at each time point. A post hoc testing using Bonferroni test will be used on each group separately if the results of the one-way repeated measures, ANOVA, is found to be statistically significant.

The level of significance will be set at *p* ≤ 0.05. Statistical analysis will be performed using IBM SPSS Software version 25 for Windows (Chicago, IL, USA).

Safety endpoints will be analyzed as summary statistics during treatment. Adverse Events (AEs) will be counted once only for a given participant), presented by severity, frequency, and relationship of AEs with additional information regarding cumulative dosing. Adverse events leading to premature discontinuation from the study intervention and serious treatment-emergent AEs will be presented either in a table or a listing.

## Discussion

The therapeutic effects of curcumin have been studied in asthmatic patients in the past albeit with mixed results. This study will utilize higher doses of curcumin than what has been previously studied to amplify the effects of curcumin [9, 14]. Although it is expected that higher doses of curcumin will improve results, the oral bioavailability of curcumin has been shown to be limited due to the hydrophobic nature of curcuminoids which has been shown to cause rapid metabolism and limited uptake [18]. Recent studies have shown that the bioavailability of curcumin can be improved with intravenous administration, which was demonstrated in in vivo rat models [19]. However, the purpose of this study is to evaluate an adjunctive therapy that could be purchased over the counter. Therefore, black pepper extract (active ingredient: piperine) was added to the formulation due to the fact that when curcumin is given concomitantly with piperine, the bioavailability of curcumin has been shown to increase by 2000%; an effect we hope will improve outcomes when compared to prior studies [20]. The higher dosage will be achieved by having patients self-administer curcumin on a twice-daily schedule. A potential issue that may arise because of this design is that self-administration of the curcumin may lead to inaccurate administration (e.g., missed doses) due to limitations of oversight and the inability to test serum curcumin levels. Furthermore, the twice-daily schedule may lead to reduced adherence as compared to a once-daily dosing schedule as has been demonstrated by prior studies [21].

Due to a limited number of available participants, this study does not exclude participants that smoke cigarettes. In previous studies researchers identified that cigarette smoking and environmental pollutants can lead to the activation of the NF-kB pathway [22]. By including participants that smoke cigarettes the benefits of curcumin may be skewed.

With the onset of the COVID-19 pandemic, asthmatics have become even more vulnerable and have been found to be especially susceptible to more severe COVID-19 infections. As a result, this study has not been able to take place as was previously scheduled due to on-going safety concerns regarding the initial encounter because patients will need to be seen in clinic rather than via telecommunications which have predominated since the onset of the pandemic. However, it is important to recognize that the pandemic has spurred physicians to think outside the box for medical management by exploring the benefits of various cost-effective supplements, such as Vitamin D and curcumin. In fact, curcumin has been shown to inhibit the angiotensin-converting enzyme2 (ACE-2) pathway within the lungs, and as such, has been theorized to protect against the development of acute respiratory distress (ARDS) or acute lung injury (ALI), two lung processes that are commonly associated with lethal COVID-19 infections [23]. Therefore, we hope our study will shed light on the utility of curcumin and its role in reducing lung inflammation.

## Data Availability

Not applicable.

## Abbreviations

ACE-2: Angiotensin-converting enzyme2
AE: Adverse event
ALI: Acute lung injury
ARDS: Acute respiratory distress
COVID-19: Corona virus disease
COX-2: Cyclooxygenase-2
Der f: Dermatophagoides farinae
FeNO: Nitric oxide
FEV1: Forced exhaled volume in 1 s
FVC: Forced vital capacity
GINA: Global initiative for asthma
HDAC: Histone deacetylase
ICS: Inhaled corticosteroids
IDS: Intrusion detection system
IgE: Immunoglobulin E
IL-6: Interleukin-6
INR: International normalized ratio
LABA: Long-acting beta agonist
LAMA: Long acting muscarinic
LTRA: Leukotriene receptor antagonist
MAPK: Mitogen-activated protein kinase
NF-_k_B: Nuclear factor kappa B
Nrf2/HO-1: Nuclear factor erythroid 2-related factor 2/heme oxygenase 1
PT: Prothrombin time
PTT: Partial thromboplastin time
TNF-α: Tumor necrosing factor-alpha
V1: Baseline visit
V2-4: Monthly clinical visits
